# The impact of folding modes and deuteration on the atomic resolution structure of hen egg-white lysozyme

**DOI:** 10.1107/S2059798321010950

**Published:** 2021-11-17

**Authors:** Joao Ramos, Valerie Laux, Michael Haertlein, V. Trevor Forsyth, Estelle Mossou, Sine Larsen, Annette E. Langkilde

**Affiliations:** a Life Sciences Group, Institute Laue–Langevin, 71 Avenue des Martyrs, 38000 Grenoble, France; b Partnership for Structural Biology (PSB), 71 Avenue des Martyrs, 38000 Grenoble, France; c Department of Drug Design and Pharmacology, University of Copenhagen, Universitetsparken 2, DK-2100 Copenhagen, Denmark; d Faculty of Natural Sciences, Keele University, Newcastle ST5 5BG, United Kingdom; e Faculty of Medicine, Lund University, 221 00 Lund, Sweden; f LINXS Institute for Advanced Neutron and X-ray Science, Scheelvagen 19, 223 70 Lund, Sweden; g European Synchrotron Radiation Facility, 71 Avenue des Martyrs, 38000 Grenoble, France; h Department of Chemistry, University of Copenhagen, Universitetsparken 5, DK-2100 Copenhagen, Denmark

**Keywords:** *in vitro* refolding, isotope effect, thermal stability, folding modes, deuteration, folding dynamics, hen egg-white lysozyme, enzymatic activity, X-ray crystallography

## Abstract

The biological function of a protein is intimately related to its structure and dynamics, which in turn are determined by the way in which it has been folded. *In vitro* refolding is commonly used for the recovery of recombinant proteins that are expressed in the form of inclusion bodies and is of central interest in terms of the folding pathways that occur *in vivo*. Here, biophysical data are reported for *in vitro*-refolded hydrogenated hen egg-white lysozyme, in combination with atomic resolution X-ray diffraction analyses, which allowed detailed comparisons with native hydrogenated and refolded perdeuterated lysozyme. Distinct folding modes are observed for the hydrogenated and perdeuterated refolded variants, which are determined by conformational changes to the backbone structure of the Lys97–Gly104 flexible loop. Surprisingly, the structure of the refolded perdeuterated protein is closer to that of native lysozyme than that of the refolded hydrogenated protein. These structural differences suggest that the observed decreases in thermal stability and enzymatic activity in the refolded perdeuterated and hydrogenated proteins are consequences of the macromolecular deuteration effect and of distinct folding dynamics, respectively. These results are discussed in the context of both *in vitro* and *in vivo* folding, as well as of lysozyme amyloidogenesis.

## Abstract 2

A study of *in vitro* refolding and isotope effects on protein structure, activity and stability shows that different folding dynamics can lead to important changes in protein properties.

## 1. Introduction

Secreted eukaryotic proteins produced *in vivo* typically pass through a process that starts at the ribosome (for example, at the endoplasmic reticulum). The initial stages of translation involve the production of a pre-sequence or signal peptide that binds to the signal recognition particle (SRP), which in turn binds to the membrane-bound SRP receptor. The unfolded peptide then passes through the membrane, whereupon the pre-sequence is cleaved, with folding subsequently occurring through a pathway involving multiple chaperones (Shikano & Colley, 2013 ▸). In the case of hen egg-white lysozyme (HEWL), recombinant production in *Escherichia coli* is problematic because the reductive environment of the bacterial cytosol prevents the correct formation of the four disulfide bridges, resulting in the formation of inclusion bodies. For the production of perdeuterated protein, we have recently adopted an approach whereby large quantities of insoluble protein were produced as inclusion bodies in *E. coli* followed by an optimized *in vitro* refolding process (Ramos *et al.*, 2021 ▸).

HEWL has been shown to possess two distinct folding domains (Supplementary Fig. S1): the α-domain, constituted by four α-helices, the Val120–Arg125 3_10_-helix and the loops Gly16–Gly22 and Ser100–Ala107, and the β-domain, which comprises a triple-stranded antiparallel β-sheet, the Pro79–Leu84 3_10_-helix and the Arg61–Ile78 loop (Miranker *et al.*, 1991 ▸). While the α-domain, which contains most of the secondary-structure motifs, folds first, the β-domain is stabilized later in the folding process (Miranker *et al.*, 1991 ▸, 1993 ▸; Radford *et al.*, 1992 ▸). The *in vitro* folding mechanism of lysozyme was later shown to involve intermediate states (Radford *et al.*, 1992 ▸; Miranker *et al.*, 1993 ▸; Wildegger & Kiefhaber, 1997 ▸). Additionally, the unfolding process of an amyloidogenic variant of human lysozyme, highly homologous to HEWL, seems to involve local cooperativity (Canet *et al.*, 2002 ▸). An earlier study had also suggested identity between the unfolding and folding intermediates of lysozyme and its structural homologue α-lactalbumin (Ikeguchi *et al.*, 1986 ▸). Understanding protein folding and the impact of different chemical environments on folding pathways is essential for current efforts in predicting three-dimensional structure using *in silico* methods and for the study of amyloidogenic pathologies. In the case of human lysozyme, several amyloidogenic mutations have been identified, particularly in α-helix C (Ile88–Asp101) and the β-domain (Pepys *et al.*, 1993 ▸; Gillmore *et al.*, 1999 ▸; Valleix *et al.*, 2002 ▸; Yazaki *et al.*, 2003 ▸; Wooliver *et al.*, 2007 ▸; Girnius *et al.*, 2012 ▸; Jean *et al.*, 2014 ▸; Sperry *et al.*, 2016 ▸; Nasr *et al.*, 2017 ▸).

H atoms constitute approximately 50% of all atoms in a protein. They are essential to biological function, either through their role in protein folding, in protein interactions or by participating in the catalysis of enzymatic reactions. While the substitution of H by its heavier isotope deuterium (D) in solvent-exchangeable positions (*i.e.* those bound to N or O atoms) or at all positions (*i.e.* perdeuteration) usually results in closely isomorphous structures, the associated mass difference can be expected to have consequences for protein dynamics. This isomorphism is routinely used in neutron macromolecular crystallography (NMX; Cuypers *et al.*, 2013 ▸; Manzoni *et al.*, 2018 ▸; Yee *et al.*, 2019 ▸; McGregor *et al.*, 2021 ▸; Gajdos *et al.*, 2021 ▸; Kelpšas *et al.*, 2021 ▸), small-angle neutron scattering (SANS; Breyton *et al.*, 2013 ▸; Maric *et al.*, 2014 ▸, 2015 ▸; Dunne *et al.*, 2017 ▸; Nitsche *et al.*, 2018 ▸; Kehlenbeck *et al.*, 2019 ▸), neutron reflectometry (NR; Wacklin *et al.*, 2016 ▸; Moulin *et al.*, 2018 ▸; Campbell *et al.*, 2018 ▸; Waldie *et al.*, 2018 ▸, 2019 ▸), neutron spectroscopy (Foglia *et al.*, 2019 ▸), neutron fiber diffraction (Gardner *et al.*, 2004 ▸; Wada *et al.*, 2011 ▸) and nuclear magnetic resonance (NMR; Varga *et al.*, 2007 ▸), where the different physical properties of the two isotopes are fully exploited. One of the common ways of testing this isomorphism is through a comparison of the X-ray analyses of analogous structures, and there is now a steadily growing database of these comparisons. For crystalline systems, the isomorphism usually holds good to high resolution (Gamble *et al.*, 1994 ▸; Cooper *et al.*, 1998 ▸; Meilleur *et al.*, 2004 ▸; Artero *et al.*, 2005 ▸; Liu *et al.*, 2007 ▸; Fisher & Helliwell, 2008 ▸; Cuypers *et al.*, 2013 ▸; Yee *et al.*, 2016 ▸, 2019 ▸; Koruza *et al.*, 2019 ▸; Ramos *et al.*, 2021 ▸). For the HEWL work described here, this is, to our knowledge, the first time that a detailed comparative study has been made of hydrogenated and perdeuterated analogs of a refolded protein.

While hydrogenated and perdeuterated variants of a protein are usually close to identical in structure, several studies of the macromolecular H/D isotope effect have reported decreases in the thermal stability of perdeuterated proteins compared with their hydrogenated analogs (Berns, 1963 ▸; Hattori *et al.*, 1965 ▸; Brockwell *et al.*, 2001 ▸; Meilleur *et al.*, 2004 ▸; Koruza *et al.*, 2018 ▸; Nichols *et al.*, 2020 ▸; Ramos *et al.*, 2021 ▸). Observations have also been made regarding solvent H/D isotope effects which suggest that both perdeuterated and hydrogenated proteins are thermally more stable in D_2_O than in H_2_O (Hattori *et al.*, 1965 ▸; Harrington & von Hippel, 1961 ▸; Makhatadze *et al.*, 1995 ▸; Freyman *et al.*, 2001 ▸; Kuhlman & Raleigh, 1998 ▸; Sasisanker *et al.*, 2004 ▸; Efimova *et al.*, 2007 ▸). While the macromolecular isotope effect seems to be caused by changes in the strengths of hydrophobic interactions between residue side chains, as described by Hattori *et al.* (1965 ▸), the solvent isotope effect appears to be a consequence of variations in protein–protein and protein–water hydrogen bonds following H/D substitution in exchangeable positions and to different solvation potentials for D_2_O and H_2_O (Svergun *et al.*, 1998 ▸; Sasisanker *et al.*, 2004 ▸; Artero *et al.*, 2005 ▸; Efimova *et al.*, 2007 ▸; Jasnin *et al.*, 2008 ▸). Changes in protein solubility and crystallization conditions are also apparent (Budayova-Spano *et al.*, 2000 ▸; Hazemann *et al.*, 2005 ▸; Liu *et al.*, 2007 ▸; Petit-Haertlein *et al.*, 2009 ▸; Koruza *et al.*, 2018 ▸). Often the crystallization conditions used for the perdeuterated variant comprise reductions in the precipitant and/or protein concentration, reflecting decreases in protein solubility compared with that of the hydrogenated variant (Hazemann *et al.*, 2005 ▸; Petit-Haertlein *et al.*, 2009 ▸). Nevertheless, crystallo­graphic studies have been performed in which the crystallization conditions for both proteins are substantially different (Liu *et al.*, 2007 ▸). These variations need to be considered when discussing any structural changes associated with deuteration and when attributing these to macromolecular isotope effects.

In our previous study (Ramos *et al.*, 2021 ▸), we compared the structure of a perdeuterated refolded hen egg-white lysozyme variant (D-HEWL_EC_) with that of the native hydrogenated variant (H-HEWL). This analysis was mainly carried out in the context of establishing the viability and validity of a neutron crystallographic study, in which major technical benefits were gained through the use of perdeuterated protein. The available data did not permit the effects of macromolecular and solvent isotope substitution (H/D) to be clearly distinguished from differences associated with folding dynamics. However, protocols developed to further produce the hydrogenated refolded analog (H-HEWL_EC_) allowed a detailed comparison of the hydrogenated and perdeuterated analogs. With the aid of new X-ray crystallo­graphic data to 0.89 Å resolution for H-HEWL_EC_ (PDB entry 7p6m), along with thermal stability and activity assays, we characterize the different effects in detail. Both the H-HEWL_EC_ and D-HEWL_EC_ variants differ from native H-HEWL by an additional glycine residue at the N-terminus. This residue disrupts hydrogen-bond interactions at the N-terminus of the protein, rendering this region more dis­ordered (Ramos *et al.*, 2021 ▸), although it did not perturb protein folding or function. The observed changes in enzymatic activity have been correlated with structural differences in the three variants. Additionally, the thermal stabilities of the three variants were investigated by differential scanning fluorimetry (DSF) in hydrogenated as well as deuterated solutions. This systematic comparison enables us to address and separate the effects of *in vitro* refolding, as well as the macromolecular and solvent isotope effects.

## 2. Materials and methods

### 2.1. Protein materials

Commercially available H-HEWL (catalog No. L6876; Sigma–Aldrich) was used without additional processing. D-HEWL_EC_ was recombinantly expressed in *E. coli* BL21(DE3) cells in the form of inclusion bodies and was purified and refolded in-column as described previously (Ramos *et al.*, 2021 ▸). Using a similar approach, H-HEWL_EC_ was expressed in *E. coli* BL21(DE3) cells, purified and refolded. The buffer compositions and procedures employed in inclusion-body washing, protein purification by gel filtration and refolding in-column were identical for both proteins. The main differences between the protein-production methods used for the two HEWL_EC_ variants were the culture media used for protein overexpression and the final protein buffer exchange performed after refolding. D-HEWL_EC_ was expressed in *E. coli* cultures adapted to fully deuterated minimal medium, while H-HEWL_EC_ was produced in *E. coli* cultures grown in H_2_O minimal medium. Additionally, D-HEWL_EC_ was buffer-exchanged into a D_2_O solution of 50 m*M* sodium acetate pD 4.5 (pD = pH + 0.4; Glasoe & Long, 1960 ▸). Meanwhile, H-HEWL_EC_ was buffer-exchanged into a H_2_O solution of 50 m*M* sodium acetate pH 4.5. The proteins were concentrated in their final buffer solutions to 20 mg ml^−1^ for further experiments.

### 2.2. Differential scanning fluorimetry

Protein thermal stability was assessed by DSF using a Prometheus instrument from NanoTemper. Protein unfolding is measured through the intrinsic fluorescent signal of Trp residues (there are six Trp residues in HEWL), which is quenched when the protein is folded and is enhanced when these residues are exposed to the solvent in the unfolded state. H-HEWL, D-HEWL_EC_ and H-HEWL_EC_ were each tested in four different buffer solutions: (i) 50 m*M* sodium acetate pH 4.5 in H_2_O, (ii) 50 m*M* sodium acetate pD 4.5 in D_2_O, (iii) 0.1 *M* sodium phosphate pH 7.5, 0.1 *M* NaCl, 2 m*M* NaN_3_ in H_2_O and (iv) 0.1 *M* sodium phosphate pD 7.5, 0.1 *M* NaCl, 2 m*M* NaN_3_ in D_2_O. The samples were diluted from stock solutions at 20 mg ml^−1^ in each buffer solution to a final protein concentration of approximately 0.2 mg ml^−1^. The fluorescence was measured at 90% instrument excitation power in temperature ramps from 20°C to 95°C with increments of 0.1°C min^−1^. The results presented were obtained from three different experiments with triplicate measurements for each condition.

### 2.3. Activity assays

The enzymatic activity was measured using the method originally reported by Shugar (1952 ▸), which estimates the activity rates by following the decrease in absorbance at 450 nm when HEWL is added to a cell suspension of *Micrococcus lysodeikticus*. The conditions used were identical to those used in our previous study (Ramos *et al.*, 2021 ▸). The results presented in Supplementary Fig. S2 were obtained for all HEWL variants in three experiments, with triplicate measurements for each condition (technical replicates), and controls were included as buffer without protein. The technical replicates were averaged and plotted against time to obtain the activity curves. The initial velocities were retrieved from the linear phase (*R* ^2^ > 0.95) corresponding to the first 5 min of reaction. The final activity rates and corresponding standard deviations were obtained from averaging the results from the three experiments.

### 2.4. Protein crystallization

Triclinic H-HEWL_EC_ crystals were obtained by microseeding triclinic H-HEWL in microbatch under oil at 18°C. The crystallization drop consisted of 2.5 µl 20 mg ml^−1^ refolded H-HEWL_EC_, 2.5 µl 0.3 *M* NaNO_3_ and 50 m*M* sodium acetate pH 4.5 in H_2_O and 0.5 µl H-HEWL seeds. Crystals of approximately 0.1 mm^3^ appeared within one week.

### 2.5. X-ray diffraction data collection and processing

A triclinic crystal of H-HEWL_EC_ was flash-cooled in liquid nitrogen after soaking in a solution consisting of 30%(*v*/*v*) glycerol, 0.4 *M* NaNO_3_, 50 m*M* sodium acetate pH 4.5 in H_2_O. X-ray diffraction data at 100 K were measured on the BioMAX beamline (Ursby *et al.*, 2020 ▸) at MAX IV Laboratory. Data to 0.89 Å resolution were recorded at two different κ orientations with 180° scans to maximize reciprocal-space coverage and data completeness. Data reduction was performed using *XDS* (Kabsch, 2010 ▸) and the two 180° scans were scaled using *XSCALE* (Kabsch, 2010 ▸). The data were converted to *SHELX* format using *XDSCONV* (Kabsch, 2010 ▸) and 5% of the reflections were randomly flagged for *R*_free_ calculations.

### 2.6. Model refinement

PDB entry 7ave (Ramos *et al.*, 2021 ▸) was used as the initial model for the refinement of H-HEWL_EC_ in *SHELXL* (Sheldrick, 2015 ▸). Alternate protein residue conformations, water molecules and ions were removed from the initial model and the anisotropic ADPs were converted to isotropic ADPs. After the refinement of residue disorder, H atoms were added in idealized positions and fixed as riding atoms. Default parameters for the geometric restraints of the residues were employed and a few bond-angle outliers were allowed, rather than imposing stricter restraints, during refinement using high-resolution data. Anisotropic ADPs were refined for all non-H atoms, including water molecules and ions. Occupancies were refined for water molecules and ions with *B* factors larger than 30 Å^2^. SIMU (0.1) and XPND (0.001) restraints were removed in the last rounds of refinement.

### 2.7. Analysis of HEWL structures

The H-HEWL and D-HEWL_EC_ models (PDB entries 7avf and 7ave, respectively; Ramos *et al.*, 2021 ▸) were used in comparisons with the H-HEWL_EC_ structure. The structural alignments between the HEWL structures were performed using *GESAMT* from the *CCP*4 suite (Winn *et al.*, 2011 ▸). The alignments of the Lys97–Gly104 and binding-cleft regions were performed in *PyMOL* (version 2.0; Schrödinger) using the *ALIGN* function with zero refinement cycles. All illustrations of protein structures were made using *PyMOL*.

## 3. Results

### 3.1. *In vitro* refolding has a stronger impact on the thermal stability of lysozyme than deuteration

The results from the DSF experiments show clear trends regarding the effects on protein thermal stability of *in vitro* refolding, protein perdeuteration, H/D solvent substitution and the pH of the buffer solution. The melting temperatures for H-HEWL and D-HEWL_EC_ in sodium acetate pD 4.5 in D_2_O and in sodium phosphate pH 7.5 in H_2_O were remeasured in this study and are in agreement with the values reported in our previous work (Ramos *et al.*, 2021 ▸).

**Figure 1:**
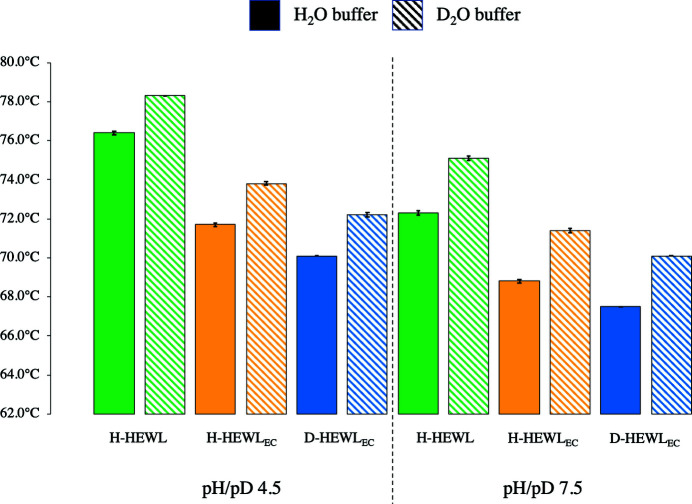
Melting temperatures measured in the DSF experiments for H-HEWL, H-HEWL_EC_ and D-HEWL_EC_ in buffer solutions at pH 4.5 and 7.5 in H_2_O and at pD 4.5 and 7.5 in D_2_O. The error bars correspond to the standard deviations of the measurements performed in three DSF experiments.

As shown in Fig. 1 ▸ and Supplementary Table S1, H-HEWL_EC_ displays a decrease in melting temperature (*T*_m_) in H_2_O of 4.7°C and 3.5°C at pH 4.5 and 7.5, respectively, in comparison with H-HEWL. In D_2_O, H-HEWL_EC_ is less thermally stable than H-HEWL by 4.5°C and 3.7°C at pD 4.5 and 7.5, respectively. The reductions in *T*_m_ seem to be constant regardless of the solvent isotope substitution, with minor variations likely to be due to the differences between pH and pD (pD = pH + 0.4; Glasoe & Long, 1960 ▸).

Perdeuteration of HEWL_EC_ appears to reduce the protein thermal stability by 1.6°C and 1.3°C at pH 4.5 and pH 7.5, respectively. Interestingly, these variations are smaller than those observed between H-HEWL and H-HEWL_EC_, highlighting that the refolding process has a stronger impact on protein thermal stability than perdeuteration. Additionally, the differences in *T*_m_ are similar in H_2_O and D_2_O solutions, reflecting effects of protein perdeuteration rather than solvent isotope substitution.

Comparing the individual variants in H_2_O and D_2_O respectively, as shown in Fig. 1 ▸, the solvent isotope effect has similar magnitudes across the three HEWL proteins, varying only according to the buffer solution pH/pD. In the case of H-HEWL, the replacement of H_2_O with D_2_O increased its thermal stability by 1.9°C and 2.8°C at pH/pD 4.5 and 7.5, respectively. Both H-HEWL_EC_ and D-HEWL_EC_ were found to be more thermally stable in D_2_O than in H_2_O, with identical variations in *T*_m_ of 2.1°C at pH/pD 4.5 and 2.6°C at pH/pD 7.5.

The results suggest that protein thermal stability is influenced by pH. However, since the buffer solutions at pH/pD 4.5 and 7.5 differ significantly in composition, it is not appropriate to perform a direct comparison of the pH/pD effects. Nevertheless, it is clear that the thermal stability is significantly reduced for the three HEWL variants by the increase in pH/pD from 4.5 to 7.5.

### 3.2. *In vitro* refolding leads to loss of enzymatic activity

Previous studies have reported reductions in the enzymatic activity of refolded HEWL compared with the native variant (Batas & Chaudhuri, 1996 ▸; Batas *et al.*, 1999 ▸). However, no structural explanations were provided for these observations. Here, we report activity assays performed in parallel on all three HEWL variants. The results show that H-HEWL_EC_ and D-HEWL_EC_ retain 66% and 69% of the activity of H-HEWL, respectively (Supplementary Fig. S2). In our previous work, we reported an activity of 51% for D-HEWL_EC_ (Ramos *et al.*, 2021 ▸) based on an analysis of the first 8 min of reaction for the extrapolation of initial velocities, resulting in *R* ^2^ > 0.91. In this study, the first 5 min were used in the subsequent analysis, resulting in *R* ^2^ > 0.95.

### 3.3. The protein fold is retained despite *in vitro* refolding and perdeuteration

The conditions used to obtain the triclinic H-HEWL_EC_ crystals were virtually identical to those reported for H-HEWL and D-HEWL_EC_ (PDB entries 7avf and 7ave, respectively; Ramos *et al.*, 2021 ▸) and comparable X-ray diffraction data extending to 0.89 Å resolution were collected at 100 K. The data-merging and model-refinement statistics are shown in Table 1 ▸. The HEWL crystal structure reported by Wang *et al.* (2007 ▸) obtained from X-ray diffraction data to 0.65 Å resolution stands out as a reference among the plethora of HEWL structures deposited in the PDB (Berman *et al.*, 2000 ▸). However, a detailed comparison of the structures of several HEWL variants should only encompass those obtained using highly similar conditions for protein crystallization, X-ray data collection, processing and model refinement.

**Figure 2:**
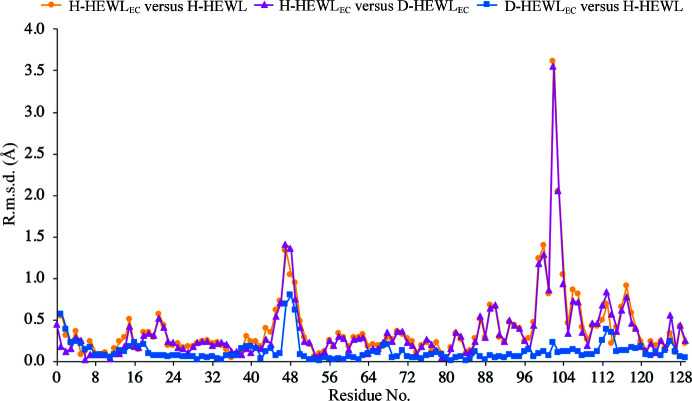
Pairwise structural comparisons. The structures were aligned using *GESAMT* from the *CCP*4 suite (Winn *et al.*, 2011 ▸) and the r.m.s.d. between each pair of structures is plotted on the residue level.

Structural alignments were thus performed for the following pairs of crystal structures: H-HEWL–H-HEWL_EC_ (highlighting refolding effects), D-HEWL_EC_–H-HEWL_EC_ (highlighting deuteration effects) and H-HEWL–D-HEWL_EC_ (combining the effects previously observed). Overall, considering H-HEWL as a reference, the protein fold is retained in both recombinant variants, despite the *in vitro* refolding that was performed as well as the additional perdeuteration of D-HEWL_EC_ (Fig. 2 ▸). This evidence is supported by the fact that the two HEWL_EC_ variants could be successfully crystallized in highly comparable conditions, in an identical space group and with similar unit-cell dimensions. Interestingly, H-HEWL and D-HEWL_EC_ are highly similar structurally and the main differences between the protein structures are observed in relation to H-HEWL_EC_. While the r.m.s.d. of D-HEWL_EC_ from the H-HEWL structure is only 0.17 Å, H-HEWL_EC_ shows an r.m.s.d. of 0.56 Å. Additionally, H-HEWL_EC_ was found to have an r.m.s.d of 0.55 Å when compared with D-HEWL_EC_. As illustrated in Fig. 2 ▸, the main differences between H-HEWL_EC_ and the two other HEWL variants are observed in the Thr47–Gly49 and Lys97–Gly104 regions, both of which are at the protein surface and engage in crystal contacts.

### 3.4. Refolded H-HEWL_EC_ appears to be more compact and rigid than native H-HEWL

47 and 51 water molecules in the crystal structures of H-HEWL_EC_ and H-HEWL, respectively, are involved in important hydrogen-bond interactions that maintain the protein fold (*i.e.* are involved in two or more hydrogen bonds to protein atoms from nonconsecutive residues). While 34 of these water molecules are conserved in both structures, the absence of the remaining water molecules in either variant does not appear to affect their fold. The protein region where the water structures differ the most is the disordered Lys97–Gly104 loop. Differences in alternate conformations of side chains of residues such as Glu7, Arg68, Thr89 and Gln121 also promote changes to the location of structural waters without significantly perturbing the protein fold. For instance, the hydrogen-bond interactions facilitated by water molecules 314 and 387 in H-HEWL_EC_ are enabled in H-HEWL by different side-chain conformations of Glu7 and Asn44. Concomitantly, water 138 in H-HEWL is involved in identical hydrogen bonds to those established by Gly0 in H-HEWL_EC_.

104 additional water molecules were observed in the hydration shells of H-HEWL, compared with 100 in H-HEWL_EC_. This minor difference in the number of hydration water molecules can be explained by the 2*F*_o_ − *F*_c_ electron-density map cutoff at 1.5σ that was used to model structural waters. The fact that water molecules with weaker densities were not included in the models might have precluded differences in hydration and surface area between H-HEWL and H-HEWL_EC_. However, calculation of the molecular surface areas of both structures yielded values of 15 720 and 14 961 Å^2^ for H-HEWL and H-HEWL_EC_, respectively. As previously noted, H-HEWL_EC_ contains an additional Gly residue at the N-terminus compared with native H-HEWL. However, when Gly0 is removed from the structure the calculated molecular surface of H-HEWL_EC_ is only marginally affected (14 899 Å^2^).

The models of H-HEWL and H-HEWL_EC_ include 41 and 36 residues with alternate conformations, respectively (Supplementary Fig. S3). It is notable that although the resolution of the X-ray data is lower for H-HEWL (1.0 Å) than for H-HEWL_EC_ (0.89 Å), the latter displays less disorder of its protein residues and also a reduced number of structural waters, as shown previously. These discrepancies could be explained by differences in quality and completeness between the data sets; however, in this case both data sets are similar (*R*_p.i.m._ of ∼5% and completeness of >95%). An example of the reduced flexibility of H-HEWL_EC_ is observed in the Arg112–Arg114 region, where both Arg residues display single conformations. In H-HEWL these Arg residues adopt alternate conformations, which apparently cause significant displacement of the protein backbone structure. Corroborating the decreased flexibility of H-HEWL_EC_ is the systematically lower mean residue *B* factor verified throughout the structure in comparison with that of H-HEWL (Supplementary Fig. S4). 74% of H-HEWL_EC_ residues display lower mean *B* factors for their main-chain atoms compared with H-HEWL, with exceptions being found in the following regions: Thr43–Asp48, Leu75–Ile78, Ser86–Cys94, Lys97–Trp108 and Gly126–Leu129.

### 3.5. The Lys97–Gly104 loop is crucial to HEWL folding

Our previous study revealed that the structure of the Lys97–Gly104 loop was the most affected upon *in vitro* refolding of D-HEWL_EC_. The backbone disorder of the Lys97–Gly104 region was shown to be associated with the occurrence of an Asn103 peptide-plane flip (for example in PDB entry 2vb1; Wang *et al.*, 2007 ▸). While a partial Asn103 peptide-plane flip has been described in H-HEWL with a refined occupancy of 33%, it was found that this alternate conformation was present in 46% of the molecules of D-HEWL_EC_ (Ramos *et al.*, 2021 ▸).

**Figure 3:**
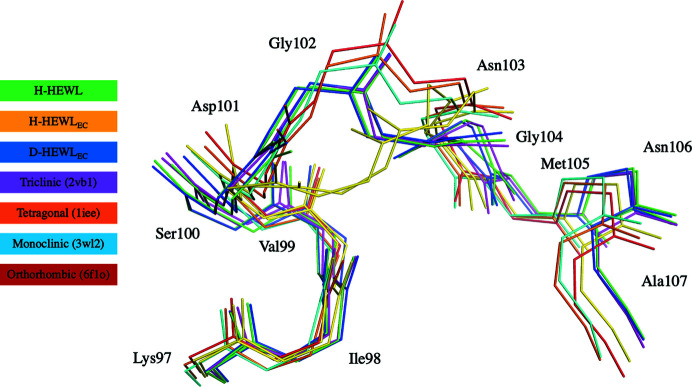
The backbone structure of the H-HEWL_EC_ Lys97–Gly104 loop (extended to Ala107) is considerably different from those of D-HEWL_EC_, H-HEWL and of HEWL in different crystal systems. PDB entries 2vb1, 1iee, 3wl2 and 6f1o were chosen as representative of native H-HEWL crystallized in the triclinic, tetragonal, monoclinic and orthorhombic systems, respectively. The Lys97–Ala107 regions of the different variants were structurally aligned; for clarity, only the main-chain atoms are shown. This illustration was produced using *PyMOL* (version 2.0; Schrödinger).

The conformation of the Lys97–Gly104 loop in H-HEWL_EC_ differs significantly from its conformations in H-HEWL and D-HEWL_EC_ (Fig. 3 ▸). The r.m.s.d.s between this region of H-HEWL_EC_ and the same residues in H-HEWL and D-HEWL_EC_ are 2.38 and 2.33 Å, respectively, noting that the r.m.s.d. between the latter two is only 0.27 Å. Furthermore, according to the 2*F*_o_ − *F*_c_ electron-density map of H-HEWL_EC_, both conformations of this loop are associated with a peptide-plane flip of Asn103 (Supplementary Fig. S5). Variations in side-chain conformations can also be observed, namely for Asp101 and Asn103. In the case of the Asn103 side chain, significant disorder is evident from the lack of density in the 2*F*_o_ − *F*_c_ electron-density map (contoured at 1σ). Meanwhile, for Asp101 the electron density suggests a different side-chain position compared with other HEWL variants (Ramos *et al.*, 2021 ▸), leading to the disruption of a hydrogen-bond crystal contact with Glu7.

Because crystal packing can perturb protein structure, particularly at its surface (as is the case for the Lys97–Gly104 loop), it is important to compare the triclinic structures with models of HEWL obtained in different space groups (Fig. 3 ▸). The Lys97–Gly104 loop appears to be disordered, regardless of the HEWL crystal system. This flexibility is promoted by the presence of Gly102 and Gly104, which increase the rotational freedom about the peptide bonds. Common to the monoclinic (PDB entry 3wl2), tetragonal (PDB entry 1iee; Sauter *et al.*, 2001 ▸) and orthorhombic (PDB entry 6f1o; Plaza-Garrido *et al.*, 2018 ▸) structures, Asn103 of native H-HEWL seems to be flipped, whereas in the triclinic system this flip is only partial, with the major conformation being the *trans* peptide bond. Superposition of these HEWL structures reveals that their main deviations are seen for residues Asp101–Gly104 (Fig. 3 ▸). While the triclinic HEWL models containing the partial peptide flip of Asn103 seem to be influenced by the crystal contact between the Asp101 and Glu7 side chains, the H-HEWL_EC_ structure lacks this interaction, similar to other HEWL crystal systems. This observation suggests that in solution the Lys97–Gly104 loop adopts a conformation closer to that seen in the monoclinic, tetragonal and orthorhombic structures, and one could therefore expect that H-HEWL_EC_ would adopt a similar arrangement. However, it is clear that its Lys97–Gly104 loop deviates significantly from the expected conformation, particularly between Asp101 and Gly104. Furthermore, in H-HEWL_EC_ a different main-chain hydrogen-bond pattern can be observed when compared with the other native H-HEWL models. While this loop is stabilized by an Asp101 N(H)–Ile98 O hydrogen bond in monoclinic, tetragonal and orthorhombic H-HEWL, in H-HEWL_EC_ this interaction is replaced by a Gly102 N(H)–Ile98 O hydrogen bond. Additionally, an unusual Gly104 N(H)–Gly102 O hydrogen bond further stabilizes the Lys97–Gly104 loop of H-HEWL_EC_. It is noteworthy that H-HEWL in other crystal systems contains a water molecule (W312 in PDB entry 3wl2, W1010 in PDB entry 1iee and W316 in PDB entry 6f1o) that plays an important role in shaping this loop by mediating hydrogen-bond interactions between Val99 O, Gly102 N(H) and Gly104 N(H) (Supplementary Fig. S6). In the triclinic form of H-HEWL this water molecule is displaced and is only present when the Asn103 peptide plane is flipped; it no longer interacts with Gly102 N(H), which instead hydrogen bonds to Lys97 O.

Since X-ray structures can contain artifacts when studied at cryogenic temperatures (Halle, 2004 ▸), room-temperature models of native H-HEWL were also considered, namely the triclinic PDB entry 4lzt (Walsh *et al.*, 1998 ▸) and the tetragonal PDB entry 1bwj (Dong *et al.*, 1999 ▸). In the triclinic HEWL, a configuration of the Lys97–Gly104 loop similar to those of our native H-HEWL and D-HEWL_EC_ can be observed. Regarding the tetragonal model, the same water molecule (W148) as in the monoclinic, tetragonal and orthorhombic 100 K structures can be found hydrogen-bonding to Val99 O, Gly102 N(H) and Gly104 N(H) (Supplementary Fig. S6). These observations support the assumption that the structural changes present in our H-HEWL_EC_ are not artifacts from collecting X-ray data at cryogenic temperatures but rather reflect differences in the protein folding.

As noted above, in triclinic H-HEWL the Glu7 side chain is involved in crystal contacts with the Lys97–Gly104 loop through the Asp101 O^δ2^–Glu7 O^ɛ2^ hydrogen bond. Meanwhile, in other H-HEWL crystal systems Glu7 adopts a different conformation, which is stabilized by hydrogen bonds to Lys1 N(H_3_)^ζ^, Gly4 N(H) and Val2 O, with the latter being mediated by a water molecule. For clarity, the conformation of Glu7 found in the triclinic system will be referred to as conformation *A*, while the alternate conformation in other H-HEWL systems will be named conformation *B*.

**Figure 4:**
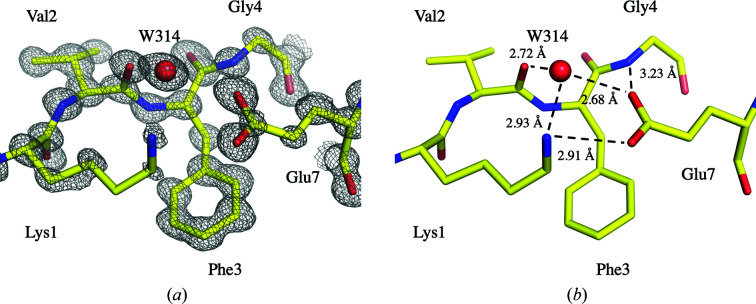
The single Glu7 conformation present in H-HEWL_EC._ The 2*F*_o_ − *F*_c_ electron-density map shown was contoured at 2σ (*a*) and the respective hydrogen-bond interactions with Lys1 N^ζ^, Gly4 N and Val2 O are depicted in (*b*). W314 mediates the interaction between Glu7 and Val2 through hydrogen bonds. This illustration was produced using *PyMOL* (version 2.0; Schrödinger).

Our triclinic H-HEWL structure displays Glu7 in conformation *A* with full occupancy. On the other hand, in D-HEWL_EC_ the Glu7 side chain is found in conformation *A* with a refined occupancy of 49% and in conformation *B* with an occupancy of 51%. Interestingly, the refined occupancies of both Glu7 and Lys97–Gly104 in conformation *B* are similar (51% and 46%, respectively). These observations indicate that the displacement of the Lys97–Gly104 loop associated with the Asn103 peptide flip is linked to the disruption of the Asp101 O^δ2^–Glu7 O^ɛ2^ hydrogen bond and the shift of the Glu7 side chain to conformation *B*. In the case of H-HEWL_EC_, where there is a greater displacement of the Lys97–Gly104 loop and where the Asp101 O^δ2^–Glu7 O^ɛ2^ hydrogen bond is completely disrupted, the Glu7 side chain is found to fully occupy conformation *B* (Fig. 4 ▸).

The conformation of Glu7 in H-HEWL_EC_ appears to be further linked to the displacement of the Thr47–Gly49 region via crystal contacts. Disorder is present in the Thr47–Gly49 region of the three HEWL variants; however, their hydrogen-bond patterns display noteworthy variations, in particular for H-HEWL_EC_ compared with both H-HEWL and D-HEWL_EC_. In H-HEWL and D-HEWL_EC_ Thr47/*A* is involved in water-mediated hydrogen bonds to Leu75 O and Glu7/*A* O^ɛ2^ via crystal contacts, while Thr47/*B* appears to establish hydrogen bonds to Leu75 O and Asn74 O. On the other hand, in H-HEWL_EC_ the fact that Glu7 is in a single conformation, identical to that of Glu7/*B* in D-HEWL_EC_, eliminates the water-mediated hydrogen bonds to Glu7/*A* O^ɛ2^ and Leu75 O via crystal contacts found in both H-HEWL and D-HEWL_EC_. Moreover, this water molecule seems to be absent in the H-HEWL_EC_ model. Therefore, while Thr47/*A* O^γ1^ participates in weak hydrogen-bond interactions with Asp48 mediated by W367, Thr47/*B* O^γ1^ can form hydrogen bonds to W365, W314 and Glu7 O^ɛ2^ via crystal contacts (Supplementary Fig. S7).

### 3.6. *In vitro* refolding perturbs the configuration of the HEWL binding cleft

**Figure 5:**
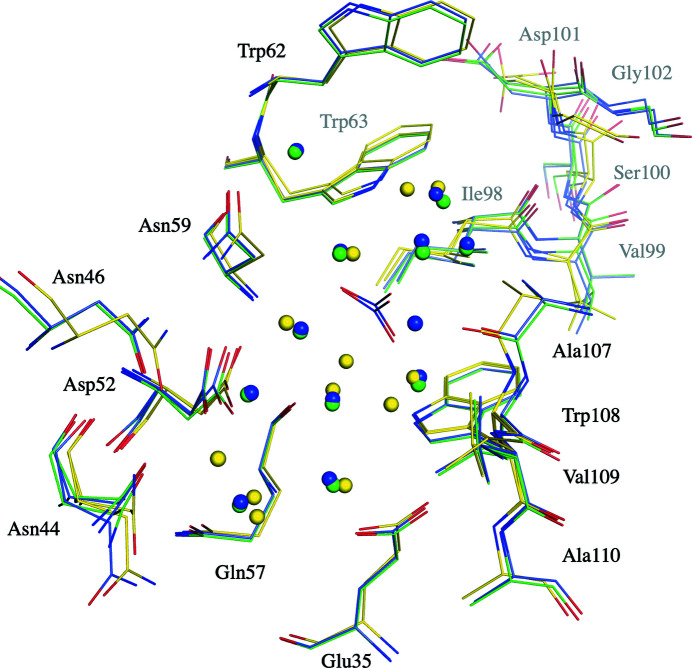
The configuration of the enzymatic binding cleft of H-HEWL_EC_ (C atoms in yellow) is altered compared with those of H-HEWL (C atoms in green) and D-HEWL_EC_ (C atoms in blue). The crystal structures of H-HEWL_EC_ and D-HEWL_EC_ were structurally aligned with that of H-HEWL and the active-site and binding-cleft residues of the enzyme are shown as sticks. The water molecules are represented as spheres colored according to the color of the respective model (*i.e.* the color of the model C atoms) and they are not labeled for clarity. The nitrate ions placed at the center of the figure belong to the D-HEWL_EC_ and H-HEWL models. This illustration was produced using *PyMOL* (version 2.0; Schrödinger).

H-HEWL_EC_ displays significant differences in the position of a number of binding-cleft residues (Fig. 5 ▸). Both Asn46 and Asp48 appear to be displaced in comparison with H-HEWL and D-HEWL_EC_, which can be explained by the crystal contacts previously described with Glu7, affecting the Thr47–Gly49 region. More importantly, the Lys97–Gly104 loop is considerably displaced, most noticeably affecting the residues Asp101–Gly104. This variation in protein structure is directly linked to the distinct hydrogen-bond pattern found in the Lys97–Gly104 loop, which stabilizes an unusual conformation of the protein backbone when compared with other H-HEWL variants. This shift in the protein main-chain structure seems to be propagated until Val109, due to the Asn106 N(H)–Asn103 O and Ala107 N(H)–Gly104 O hydrogen bonds. Additionally, displacement of the water-molecule arrangements can be observed in the active site and binding cleft of the enzyme. It is also interesting to note the absence of a nitrate ion in the H-HEWL_EC_ binding cleft which is present in the other triclinic HEWL variants (Ramos *et al.*, 2021 ▸).

An alternate conformation of Asn44 is found in H-HEWL_EC_ (Supplementary Fig. S8), which has not been observed in either triclinic H-HEWL or D-HEWL_EC_ (Ramos *et al.*, 2021 ▸) or in the 0.65 Å resolution H-HEWL structure determined by Wang *et al.* (2007 ▸). The refined occupancy of Asn44 in this conformation is 55%, where it is engaged in a 2.68 Å hydrogen bond to Gln57 O^ɛ1^. Additionally, the minor conformation, which can be found in the other triclinic HEWL variants, appears to be significantly disordered, with clear electron density only for the side-chain C^γ^ in the 2*F*_o_ − *F*_c_ electron-density map (contoured at 1σ, Supplementary Fig. S8). In addition, a fully occupied water molecule (W387) is present, forming hydrogen bonds to Asn46 O^δ1^ and the catalytic residue Asp52 O^δ2^.

## 4. Discussion

### 4.1. Deuteration and *in vitro* refolding differently affect the biophysical properties of HEWL

In this study, the contributions of *in vitro* refolding, protein perdeuteration and solvent isotope substitution to the thermal stability of HEWL have been quantified. Surprisingly, the observations suggest that *in vitro* refolding has a stronger impact on HEWL thermal stability than protein perdeuteration, with the respective decreases in *T*_m_ being greater than 3.5°C compared with variations of smaller than 1.6°C. These changes in HEWL thermal stability are consistent throughout different buffer compositions and also in H_2_O and D_2_O solvents, where the minor deviations can be explained by the aforementioned difference between pH and pD (Glasoe & Long, 1960 ▸). Moreover, the reduction in protein thermal stability associated with protein perdeuteration is in agreement with several previous studies (Berns, 1963 ▸; Hattori *et al.*, 1965 ▸; Brockwell *et al.*, 2001 ▸; Meilleur *et al.*, 2004 ▸; Koruza *et al.*, 2018 ▸; Nichols *et al.*, 2020 ▸; Ramos *et al.*, 2021 ▸).

The effects of solvent isotope substitution were measurable and of similar magnitude in all three HEWL variants. Protein thermal stability was increased by replacing the H_2_O solvent by D_2_O, with differences in *T*_m_ of between 1.9°C and 2.8°C. The small variations observed between H-HEWL and both HEWL_EC_ variants could be linked to distinct solvation structures relating to the protein-refolding process or to the addition of a Gly residue at the N-terminus of HEWL_EC_. Nevertheless, the measurement of higher protein thermal stability in D_2_O when compared with H_2_O is consistent with previous reports (Hattori *et al.*, 1965 ▸; Harrington & von Hippel, 1961 ▸; Makhatadze *et al.*, 1995 ▸; Dong *et al.*, 1997 ▸; Kuhlman & Raleigh, 1998 ▸; Sasisanker *et al.*, 2004 ▸; Efimova *et al.*, 2007 ▸). It is noted that the solvent isotope effect appears to be significantly stronger than the macromolecular isotope effect regarding protein thermal stability. This observation suggests that weakening the hydrophobic interactions of the protein residues through perdeuteration perturbs protein structure and stability to a lower degree compared with the changes in protein solvation caused by solvent isotope substitution from H_2_O to D_2_O.

It is evident that all three HEWL variants are less thermally stable in buffer solutions at pH/pD 7.5 than at pH/pD 4.5. A direct comparison of the results obtained at different pH/pD values was not possible since the buffer solutions have very different compositions. Nevertheless, since the pI of HEWL is 11, it was expected that the different variants would be less thermally stable at pH/pD 7.5 compared with a more acidic buffer solution. This observation is also consistent with the optimal pH of 5 for HEWL activity.

### 4.2. Perdeuteration can enable different folding modes of HEWL

The atomic resolution H-HEWL_EC_ crystal structure provides valuable insight regarding the effects of both *in vitro* refolding and perdeuteration on protein structure, which can be linked to differences in thermal stability and enzymatic activity. Although the protein fold is shown to be conserved in H-HEWL_EC_ (compared with H-HEWL), significant variations are observed that (as in the case of D-HEWL_EC_) stem from the Lys97–Gly104 loop. In D-HEWL_EC_ the disorder of this region seems to be favored by protein perdeuteration and *in vitro* refolding, while similar observations were made for the perdeuterated HEWL variant expressed in *Pichia pastoris*, which was produced in fully deuterated medium (Ramos *et al.*, 2021 ▸). However, it was not possible to disentangle perdeuteration and refolding effects as the structure of H-HEWL_EC_ was unknown. The slower folding dynamics caused by a combination of more viscous solvents and protein perdeuteration is likely to contribute to the increased probability of the Asn103 peptide-plane flip occurring, as well as the subsequent displacement of the Lys97–Gly104 loop (Ramos *et al.*, 2021 ▸). Unexpectedly, the structure of H-HEWL_EC_ reveals that this loop is significantly perturbed by the conditions of *in vitro* refolding. The resulting unusual Lys97–Gly104 loop conformation has important consequences for the configuration of the H-HEWL_EC_ binding cleft and for the typical crystal contacts of triclinic HEWL. Interestingly, the elution profiles of H-HEWL_EC_ and D-HEWL_EC_ during refolding show variations (Supplementary Fig. S9), which may arise from the impact of the macromolecular isotope effect in terms of mass effects on dynamics. In the case of H-HEWL_EC_ the quantities of aggregated and unfolded or misfolded protein appear to be greater, and as a consequence the refolding yield is lower in comparison with D-HEWL_EC_. This study thus revealed a greater perturbation by *in vitro* refolding than expected, and it appears that the isotope effects reduce or counteract most of these perturbations through changed dynamics.

Several crystallographic studies have provided insight into the unfolding and refolding processes of HEWL (Raskar *et al.*, 2016 ▸, 2019 ▸; Kita & Morimoto, 2016 ▸, 2020 ▸). The binding of guanidinium and urea molecules to HEWL is of particular interest since both H-HEWL_EC_ and D-HEWL_EC_ were refolded by gradually substituting a 6 *M* guanidine–HCl buffer solution with one containing 2 *M* urea. The structural changes observed in HEWL–guanidinium/urea complexes (Raskar *et al.*, 2016 ▸, 2019 ▸) are not found in any of our refolded HEWL_EC_ variants. However, an inspection of the 2*F*_o_ − *F*_c_ electron-density maps of the HEWL–urea complexes (PDB entries 5i4x, 5i54, 5i53 and 5i4y; Raskar *et al.*, 2016 ▸) revealed the potential binding of a urea molecule to the Lys97–Gly104 loop, specifically through hydrogen-bond interactions with Ile98 O, Asp101 O and Asn103 N(H) and O^δ1^. This observation suggests that the folding pathways of both H-HEWL_EC_ and D-HEWL_EC_ could be influenced by interaction with urea molecules, particularly at the Lys97–Gly104 loop, which has been shown to differ significantly from the native H-HEWL structure.

The main difference in the production of H-HEWL_EC_ and D-HEWL_EC_ is that the latter contains 698 D atoms instead of H atoms, which leads to an increase in molecular weight of 702 Da. This discrepancy has consequences for protein dynamics and importantly for the effect of dynamics on refolding. One would expect that the lighter protein (H-HEWL_EC_) would experience a faster folding process. Additionally, the strength of hydrophobic interactions, essential to protein folding, is significantly affected by isotope substitution. Hydrophobic interactions are weakened by deuteration due to the reduced vibrational amplitude of the C—D bond compared with C—H (Hattori *et al.*, 1965 ▸). The combination of the aforementioned factors suggests that the hydrophobic core of H-HEWL_EC_ would collapse faster than that of D-HEWL_EC_ and result in more stable and restrictive folding intermediates. This is likely to be the reason why H-HEWL_EC_ displays a reduced molecular surface area and fewer solvation water molecules compared with H-HEWL and D-HEWL_EC_. The formation of secondary structure through hydrogen-bond interactions is also facilitated in H-HEWL_EC_ due to the faster dynamics, permitting a larger conformational energy landscape than for the perdeuterated variant. It is at this stage that H-HEWL_EC_ would select the most favorable conformation of the Lys97–Gly104 loop, which, as shown in non-triclinic HEWL systems, involves a complete Asn103 peptide-plane flip. This conformation appears to be stabilized by unusual hydrogen bonds, including Gly102 N(H)–Ile98 O and Gly104 N(H)–Gly102 O. The fact that the Lys97–Gly104 loop is part of the HEWL α-domain reveals its importance for both protein folding and stability. In both D-HEWL_EC_ and native H-HEWL the Lys97–Gly104 loop is allowed greater flexibility, as shown by the observation of both *cis* and *trans* conformations of the Asn103 peptide bond in their crystal structures (Ramos *et al.*, 2021 ▸). Although it is assumed that peptide-plane flips occur in the early stages of protein folding and are later stabilized by hydrogen-bond formation (Hayward, 2001 ▸), HEWL crystal structures in various space groups suggest that these peptide flips are dynamical and play a role in protein flexibility. The more rigid configuration of the Lys97–Gly104 loop in H-HEWL_EC_ formed in the early stages of folding suggests a different pathway of folding than for H-HEWL and possibly even D-HEWL_EC_. Takano *et al.* (2000 ▸) have shown that a deletion mutant of Arg101 in human lysozyme (corresponding to Ser100 in HEWL) presents different folding kinetics compared with the wild-type protein. Furthermore, the rigidity of this region in H-HEWL_EC_ demonstrates its greater local structural stability, which hinders the overall plasticity of the protein and subsequently its resistance to thermal denaturation, as shown by our biophysical data.

### 4.3. The disorder of the Lys97–Gly104 region may play a role in amyloidogenesis

Interestingly, the Lys97–Gly104 loop is suggested to be involved in the formation of amyloid fibrils of HEWL. Frare *et al.* (2006 ▸) have shown that the core structure in human lysozyme amyloids encompasses the entire α-helix C and part of the β-domain (residues 32–108). Specific fragments of HEWL (Gly49–Asp101, Tyr53–Ser100, Tyr53–Asp101 and Gln57–Ala107) were found to be highly amyloidogenic, while the remaining regions of the protein remain largely soluble (Frare *et al.*, 2004 ▸). Taken alongside our structural data, these findings could indicate that HEWL α-helix C, particularly the region extending from Lys97 to Gly104, is of crucial importance in the protein-folding process and its destabilization may lead to different folding modes. This information is especially relevant in a clinical context due to the high homology between HEWL and human lysozyme. A possible link to the Lys97–Gly104 loop is seen in the amyloidogenic variants Trp64Arg and Trp112Arg of human lysozyme (corresponding to Trp63 and Trp111 in HEWL; Valleix *et al.*, 2002 ▸; Sperry *et al.*, 2016 ▸). Both Trp63 and Trp111 appear to play a crucial role in stabilizing the Lys97–Gly104 region. Trp63 enables CH–π interactions with Ile98 and the disorder of Ile98 is reflected in a similar Trp63 side-chain disorder. Meanwhile, Trp111 is part of a complex network of hydrophobic interactions that stabilize the conformation of Met105, also including Tyr23, Trp28 and Trp108.

### 4.4. The configuration of the Lys97–Gly104 loop impacts the activity of HEWL

The flexibility of the Lys97–Gly104 loop also plays a role in the activity of native HEWL, since this region is thought to be responsible for the interaction with the first three carbo­hydrate residues (A, B and C) of the substrate (Blake *et al.*, 1967 ▸; Phillips, 1967 ▸). Several X-ray crystal structures of HEWL bound to inhibitor carbohydrates have proven the importance of this region for enzymatic activity (Strynadka & James, 1991 ▸; Cheetham *et al.*, 1992 ▸; Maenaka *et al.*, 1995 ▸; Tanaka *et al.*, 2021 ▸). In particular, Asp101, which is conserved in several c-type lysozymes (Supplementary Fig. S10), is believed to make hydrogen bonds to substrate residues A and B (Blake *et al.*, 1967 ▸; Phillips, 1967 ▸). Although further clarification would require the structure of an H-HEWL_EC_–inhibitor complex, one could interpret the observed changes of the protein backbone and water structures in the binding cleft of the apo form as an indication that its ability to interact with the substrate molecule is hindered.

The observations made for the structure of H-HEWL_EC_ can provide an explanation for the reduced enzymatic activity reported for both nonrecombinant and recombinant H-HEWL in previous studies (Batas & Chaudhuri, 1996 ▸; Batas *et al.*, 1999 ▸). In the present work, H-HEWL_EC_ and D-HEWL_EC_ were refolded in identical conditions and subsequent enzymatic assays were performed for all HEWL variants, including native H-HEWL. Despite the similar levels of function of H-HEWL_EC_ and D-HEWL_EC_, the reasons for the reduction in activity by comparison with H-HEWL seem to differ according to their crystal structures. While the function of H-HEWL_EC_ appears to be hindered by differences in the configuration of the binding cleft of the enzyme, D-HEWL_EC_ activity might be primarily affected by the macromolecular isotope effect, which alters protein dynamics.

## 5. Conclusions

In summary, this study has shown that *in vitro* refolding may lead to significant changes in protein structure, affecting thermal stability and activity; these aspects are very important in the context of the folding pathways that occur *in vivo* and *in vitro*. Nevertheless, this method of protein production from inclusion bodies is an attractive approach to facilitate recombinant protein production and improve protein yields (Ramos *et al.*, 2021 ▸). Intriguingly, the perdeuterated protein was found to be closer in structure to native H-HEWL than it was to its hydrogenated variant; this indicates that the folding dynamics are different for the hydrogenated and perdeuterated analogs. These observations emphasize the significance of structural data in validating *in vitro* refolding approaches and in understanding any differences in behavior and function. Finally, the effects of deuteration (macromolecular and solvent) in protein-folding dynamics have not been extensively studied to date; however, the underlying slower dynamics of perdeuterated proteins could be of interest in mimicking the crowded cellular environment and the action of chaperones in which proteins are natively folded.

## Supplementary Material

PDB reference: hydrogenated refolded hen egg-white lysozyme, 7p6m

Supplementary Figures. DOI: 10.1107/S2059798321010950/lp5052sup1.pdf

Structure factors: contains datablock(s) H-HEWL_EC_Xray_100K_confA_39. DOI: 10.1107/S2059798321010950/lp5052sup2.hkl

## Figures and Tables

**Table 1:** X-ray diffraction data-collection and model-refinement statistics for H-­HEWL_EC_ (PDB entry 7p6m) Values in parentheses are for the outer resolution shell.

Temperature (K)	100
Source	BioMAX, MAX IV
Detector	EIGER 16M
Wavelength (Å)	0.700
Resolution range (Å)	22.46–0.89 (0.92–0.89)
Space group	*P*1
*a*, *b*, *c* (Å)	26.12, 30.70, 33.57
α, β, γ (°)	88.968, 72.768, 69.505
Total reflections	234437 (23523)
Unique reflections	68446 (6717)
Multiplicity	3.4 (3.5)
Completeness (%)	96.2 (94.3)
Mean *I*/σ(*I*)	7.4 (2.2)
*R*_merge_	0.083 (0.647)
*R*_meas_	0.098 (0.762)
*R*_p.i.m._	0.052 (0.398)
CC_1/2_	99.6 (79.9)
Reflections used in refinement with *F*_o_/σ(*F*_o_) > 4/all reflections	50427/65023
Reflections used for *R*_free_ with *F*_o_/σ(*F*_o_) > 4/all reflections	2635/3423
*R*_work_ for reflections with *F*_o_/σ(*F*_o_) > 4/all reflections	11.16/12.51
*R*_free_ for reflections with *F*_o_/σ(*F*_o_) > 4/all reflections	13.85/15.31
No. of non-H atoms (overall)	
Total	1496
Macromolecules	1304
Ligands	40
Solvent	152
Protein residues	130
R.m.s.d., bond lengths (Å)	0.023
R.m.s.d., angles (°)	3.32
Ramachandran favored (%)	95.7
Ramachandran allowed (%)	4.3
Ramachandran outliers (%)	0
Rotamer outliers (%)	3.6
Clashscore	8
Average *B* factor (Å^2^)	
Overall	14.7
Macromolecules	14.0
Ligands	15.0
Solvent	20.9
