# Supplementary material for: The impact of folding modes and deuteration on the atomic resolution structure of hen egg-white lysozyme
Source: Acta Crystallogr D Struct Biol. 2021 Nov 17;77(Pt 12):1579–90. doi: 10.1107/S2059798321010950 (PMC8647175; doi:10.1107/S2059798321010950)
Supplement: Supplementary file 1 [file d-77-01579-sup1.pdf]

STRUCTURAL
BIOLOGY

Volume 77 (2021)

Supporting information for article:

The impact of folding modes and deuteration on the atomic resolution structure of hen egg-white lysozyme

Joao Ramos, Valerie Laux, Michael Haertlein, V. Trevor Forsyth, Estelle Mossou, Sine Larsen and Annette E. Langkilde

Table S1 Melting temperatures and respective standard uncertainties measured by DSF for the three HEWL variants in D_2O and H_2O buffer at pH/pD 4.5 and 7.5.

Buffers	T_m (°C)		
	H-HEWL	H-HEWL$_{EC}$	D-HEWL$_{EC}$
D_2O sodium acetate pD 4.5	78.5 ± 0.2	73.9 ± 0.1	72.2 ± 0.1
H_2O sodium acetate pH 4.5	76.4 ± 0.1	71.7 ± 0.1	69.9 ± 0.4
D_2O sodium phosphate pD 7.5	75.3 ± 0.2	71.4 ± 0.1	70.0 ± 0.2
H_2O sodium phosphate pH 7.5	72.7 ± 0.3	68.8 ± 0.1	67.3 ± 0.3

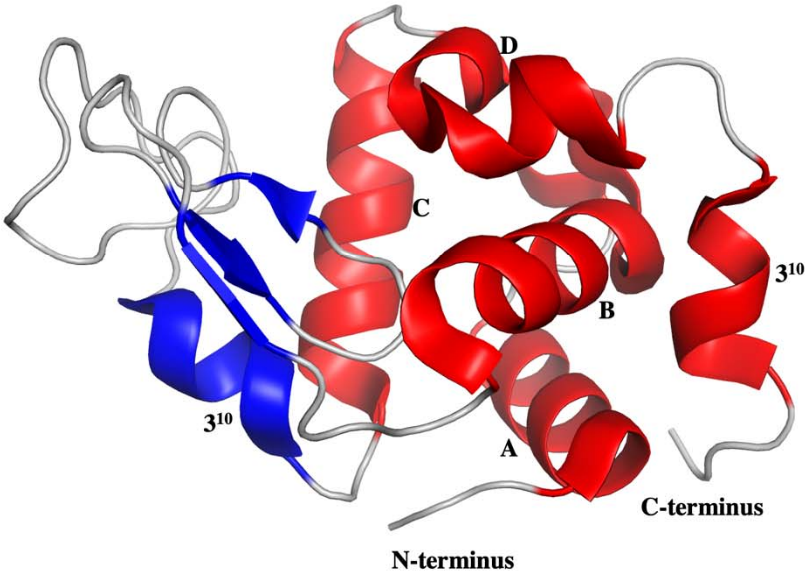

Figure S1 Cartoon representation of the native H-HEWL structure, highlighting its folding domains. The α-domain is colored in red and comprises the α-helices A-D and the Asp119-Arg125 3^{10}-helix. The β-domain is colored in blue, including the triple-stranded antiparallel β-sheet and the Pro79-Ser85 3^{10}-helix. This illustration was produced using PyMOL (version 2.0, Schrödinger).

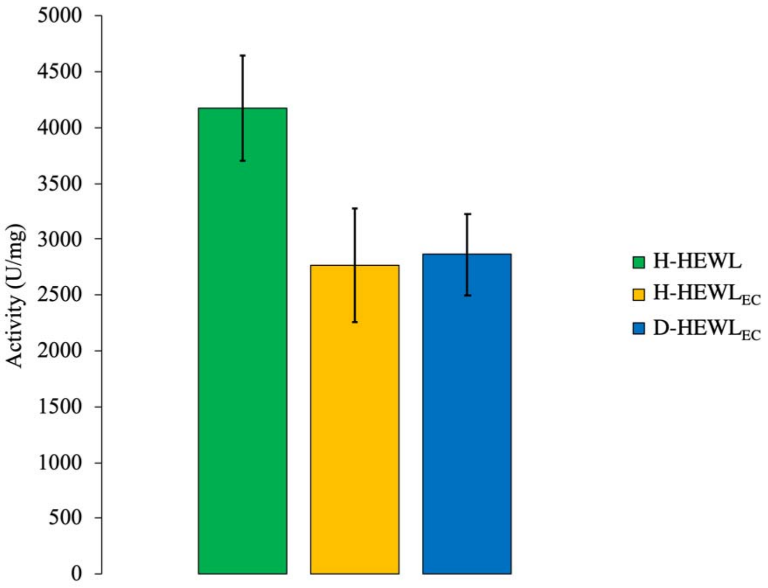

Figure S2 Measured activity rates of H-HEWL, H-HEWL$_{EC}$ and D-HEWL$_{EC}$, at 25° C, in H_2O buffer solutions at pH 7.5. H-HEWL showed an activity rate of 4172 ± 473 U/mg, while H-HEWL$_{EC}$ and D-HEWL$_{EC}$ displayed activities rates of 2765 ± 507 U/mg and 2864 ± 365 U/mg, respectively.

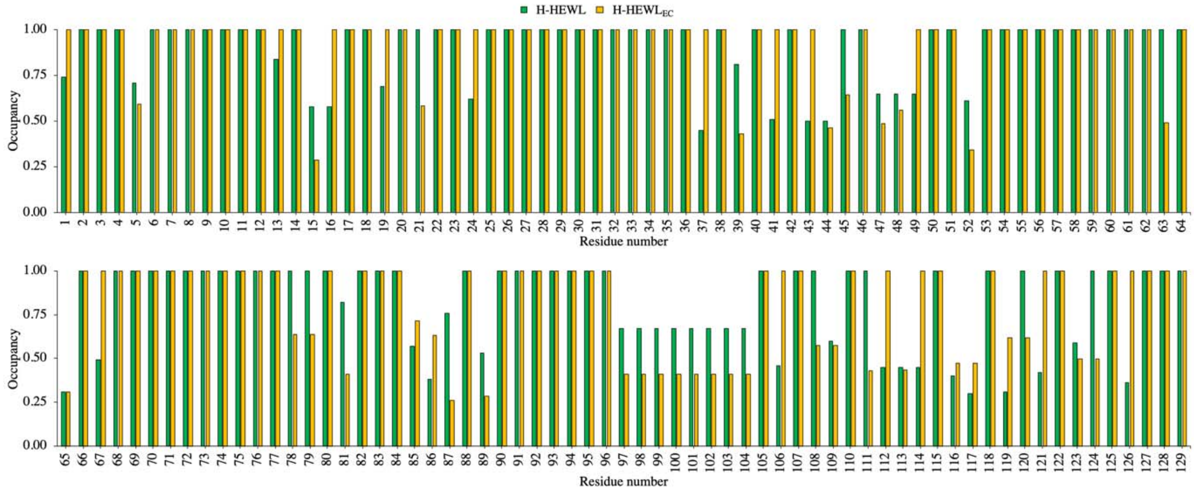

Figure S3 Refined occupancies of protein residues' conformation A for H-HEWL and H-HEWL$_{EC}$, highlighting their differences in structural disorder.

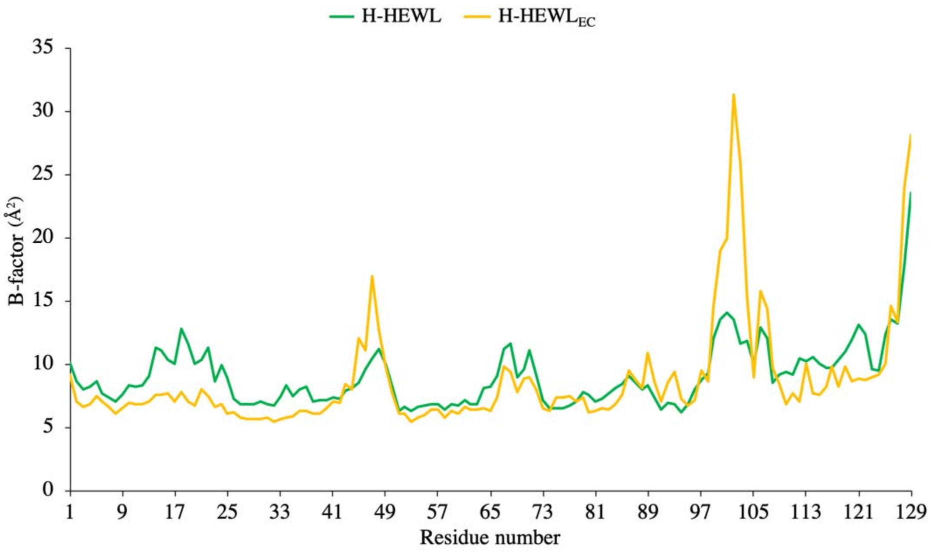

Figure S4 Mean residue B-factor for the main-chain atoms of H-HEWL and H-HEWL$_{EC}$.

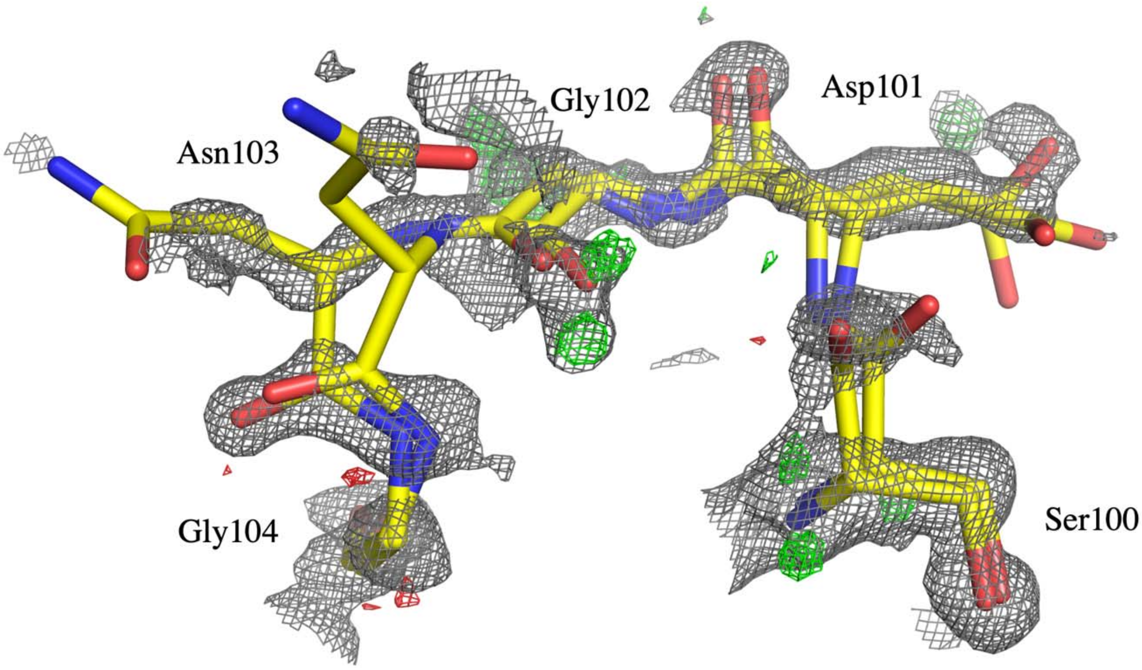

Figure S5 Backbone disorder of the Lys97-Gly104 loop and complete Asn103 peptide-plane flip in H-HEWL$_{EC}$. The 2*Fo-Fc* electron density map was contoured at 1 σ. The positive (green) and negative (red) *Fo-Fc* electrons density maps were contoured at +3 σ and -3 σ, respectively. This illustration was produced using PyMOL (version 2.0, Schrödinger).

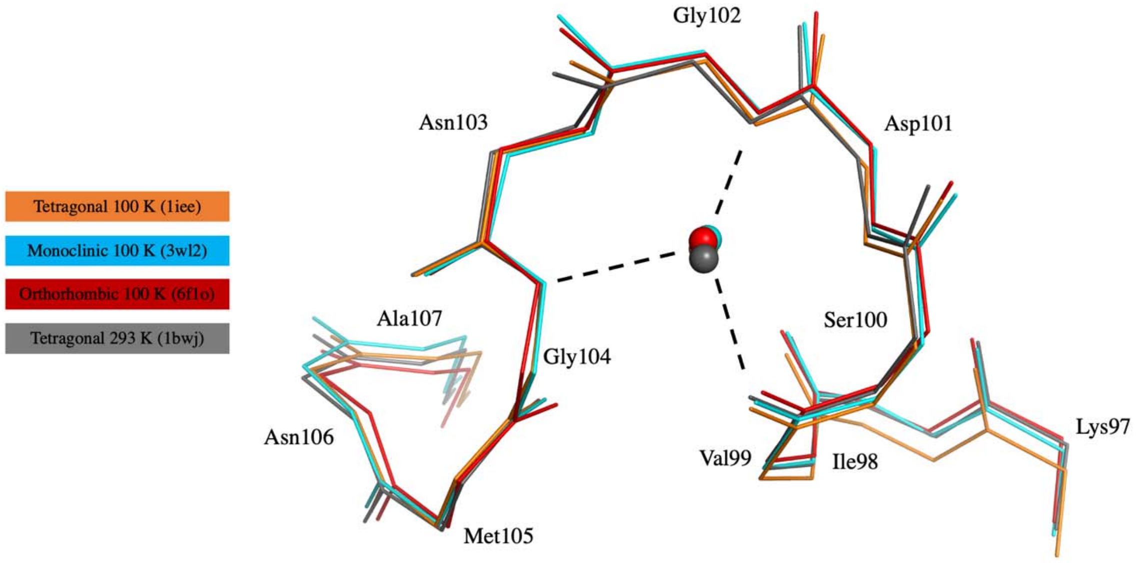

Figure S6 Localization and respective H-bond interactions of the water molecules in native H-HEWL structures (at 100 K, PDB entries 1iee, 3wl2, 6f1o, and room temperature, PDB entry 1bwj) that help stabilize the configuration of the Lys97-Gly104 loop. Water molecules 1010, 312, 316, and 148, of tetragonal 1iee, monoclinic 3wl2, orthorhombic 6f1o, and tetragonal 1bwj, respectively, are shown. Dashed lines indicate the H-bonding patterns between these water molecules and Val99 O, Gly102 N(H), and Gly104 N(H). This illustration was produced using PyMOL (version 2.0, Schrödinger).

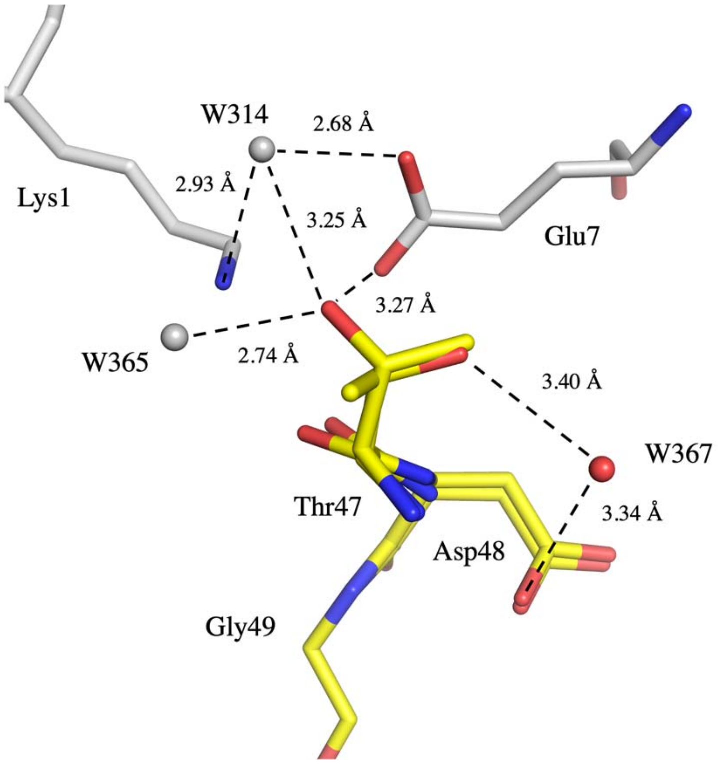

Figure S7 Representation of the H-bond interactions of Thr47 conformations A and B via crystal contacts, which promote the backbone disorder in the Thr47-Gly49 region in H-HEWL$_{EC}$. The residues (carbon atoms) and water molecules belonging to adjacent unit cells are colored in grey. This illustration was produced using PyMOL (version 2.0, Schrödinger).

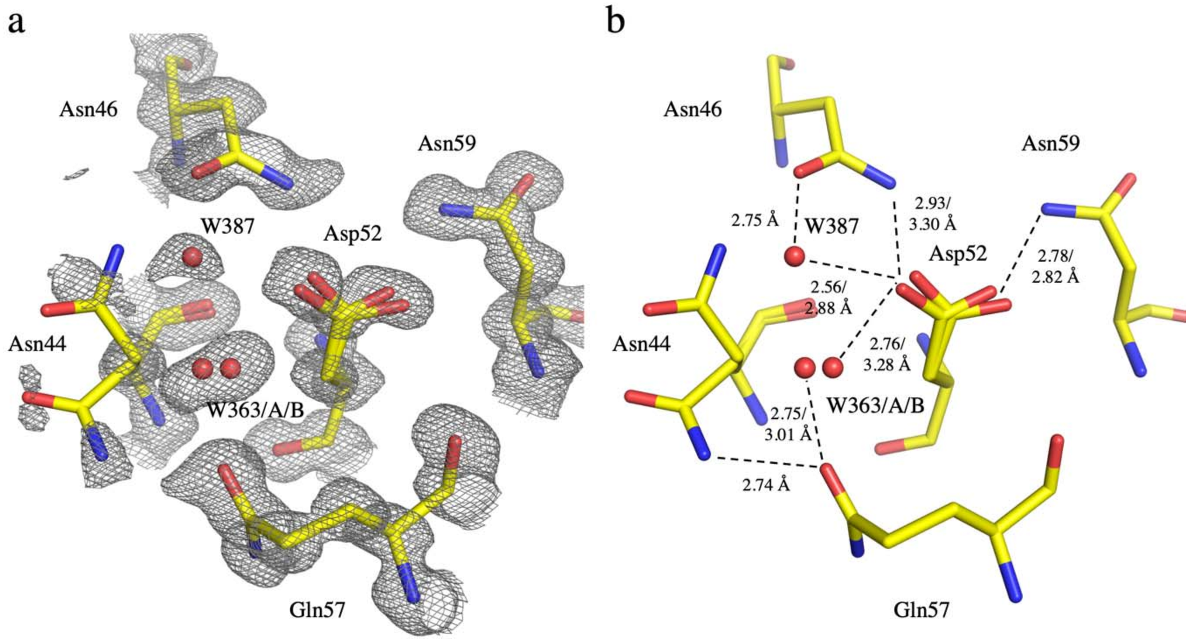

Figure S8 Alternate conformations of Asn44 and W387 found in the H-HEWL$_{EC}$ structure (a), with corresponding active site H-bond interactions (b). The 2*Fo-Fc* electron density map was contoured at 1 σ. This illustration was produced using PyMOL (version 2.0, Schrödinger).

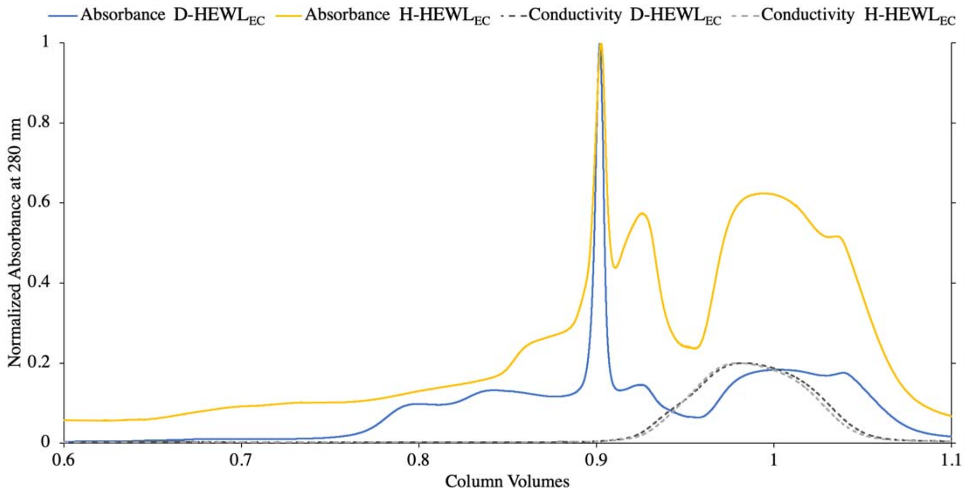

Figure S9 Superposed chromatograms of the refolding gel filtrations of D-HEWL$_{EC}$ and H-HEWL$_{EC}$.

```
Gallus gallus          1   ------------------KVFGRCELAAAMKRHGLDNYRGYSLGNWVCAAKFESNFNTQATNRNT-DGSTDYGILQINSR   61
Homo sapiens           1   MKALIVLGLVLLSVTVQGKVFERCELARTLKRLGMDGYRGISLANWMCLAKWESGYNTRATNYNAGDRSTDYGIFQINSR   80
Mus musculus           1   MKALLTLGLLLLSVTAQAKVYNRCELARILKRNGMDGYRGVKLADWVCLAQHESNYNTRATNYNRGDRSTDYGIFQINSR   80
Danio rerio            1   MRLAVVFLCLAWMSSCESKTLGRCDVYKIFKNEGLDGFEGFSIGNYVCTAYWESRFKTHRVR--SADTGKDYGIFQINSF   78
Numida meleagris       1   MKSLLILVLCFLPMAALGKVFGRCELAAAMKRHGLDNYRGYSLGNWVCAAKFESNFNSQATNRNT-DGSTDYGVLQINSR   79
Meleagris gallopavo    1   MRSLLILVLCFLPLAALGKVYGRCELAAAMKRLGLDNYRGYSLGNWVCAAKFESNFNTHATNRNT-DGSTDYGILQINSR   79

Gallus gallus         62   WWCNDGRTPGSRNLCNIPCSALLSSDITASVNCAKKIVSDGNGMNAWVAWRNRCKGTDVQAWIRGCRL       129
Homo sapiens          81   YWCNDGKTPGAVNACHLSCSALLQDNIADAVACAKRVVRDPQGIRAWVAWRNRCQNRDVRQYVQGCGV       148
Mus musculus          81   YWCNDGKTPRSKNACGINCSALLQDDITAAIQCAKRVVRDPQGIRAWVAWRTQCQNRDLSQYIRNCGV       148
Danio rerio           79   KWCDDG-TPGGKNLCKVACSDLLNDDLKASVGCAKLIVKM-DGLKSWETWDSYCNGRKMSRWVKGCEQ[7]    151
Numida meleagris      80   WWCNDGRTPGSRNLCNIPCSALQSSDITATANCAKKIVSDGNGMNAWVAWRKHCKGTDVRVWIKGCRL       147
Meleagris gallopavo   80   WWCNDGRTPGSKNLCNIPCSALLSSDITASVNCAKKIASGGNGMNAWVAWRNRCKGTDVHAWIRGCRL       147
```

Figure S10 Multiple sequence alignment of HEWL (*Gallus gallus*) with other c-type lysozymes (performed in COBALT; https://www.ncbi.nlm.nih.gov/tools/cobalt/re_cobalt.cgi), highlighting high sequence homology and the conservation of residue Asp101.
